# Photo-Healable Fabrics: Achieving Structural Control via Photochemical Solid–Liquid Transitions of Polystyrene/Azobenzene-Containing Polymer Blends

**DOI:** 10.1021/acsami.4c02578

**Published:** 2024-05-21

**Authors:** Yi-Fan Chen, Meng-Ru Huang, Yen-Shen Hsu, Ming-Hsuan Chang, Tse-Yu Lo, Bhaskarchand Gautam, Hsun-Hao Hsu, Jiun-Tai Chen

**Affiliations:** † Department of Applied Chemistry, National Yang Ming Chiao Tung University, Hsinchu 300093, Taiwan; ‡ Center for Emergent Functional Matter Science, National Yang Ming Chiao Tung University, Hsinchu 300093, Taiwan

**Keywords:** azobenzene, blend, fabric, self-healing, photoresponsive

## Abstract

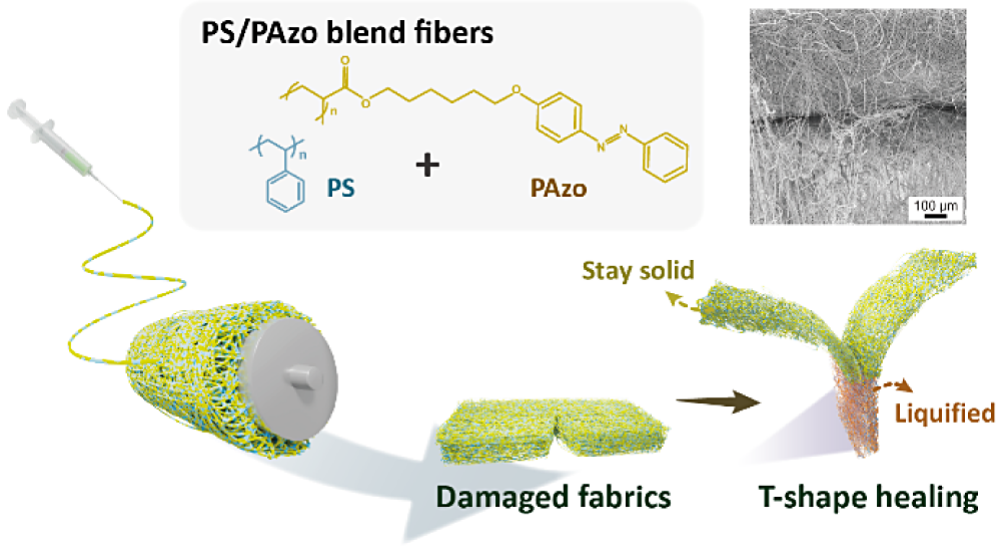

While polymer fabrics are integral to a wide range of applications, their vulnerability to mechanical damage limits their sustainability and practicality. Addressing this challenge, our study introduces a versatile strategy to develop photohealable fabrics, utilizing a composite of polystyrene (PS) and an azobenzene-containing polymer (PAzo). This combination leverages the structural stability of PS to compensate for the mechanical weaknesses of PAzo, forming the fiber structures. Key to our approach is the reversible *trans*-*cis* photoisomerization of azobenzene groups within the PAzo under UV light exposure, enabling controlled morphological alterations in the PS/PAzo blend fibers. The transition of PAzo sections from a solid to a liquid state at a low glass transition temperature (*T*_g_ ∼ 13.7 °C) is followed by solidification under visible light, thus stabilizing the altered fiber structures. In this study, we explore various PS/PAzo blend ratios to optimize surface roughness and mechanical properties. Additionally, we demonstrate the capability of these fibers for photoinduced self-healing. When damaged fabrics are clamped and subjected to UV irradiation for 20 min and pressed for 24 h, the mobility of the *cis*-form PAzo sections facilitates healing while retaining the overall fabric structure. This innovative approach not only addresses the critical issue of durability in polymer fabrics but also offers a sustainable and practical solution, paving the way for its application in smart clothing and advanced fabric-based materials.

## Introduction

Polymer fabrics have drawn significant interest because of their versatility, durability, and breathability, creating potential applications such as wearable devices,^1,2^ medical textiles,^3,4^ and smart membranes.^5,6^ The inherent softness of these materials, however, makes the materials susceptible to mechanical damage, leading to unexpected waste and inconvenience.^7,8^ To solve this issue, self-healable polymers are applied, which can repair their structures via external stimuli, including pressure,^9,10^ heat,^11,12^ chemical treatment,^13,14^ and light.^15,16^ Upon exposure to these stimuli, the physical or chemical interactions provide an opportunity to repair the morphologies of the materials. On the contrary, the high self-healing efficiency can also destroy the fiber structures, leading to a challenging balance between practicality and sustainability.^9^

Light-driven polymeric systems distinguish themselves from other external stimuli through their high spatiotemporal resolution and remote wireless controllability.^17−19^ Optically healable polymers have been developed by various mechanisms, such as noncovalent bonds,^20^ photoisomerization,^21^ metal–ligand coordination,^22^ and dynamic covalent bonds.^23^ Defined as intrinsic healable polymers, these materials without additional healing agents generally present a lower glass transition temperature (*T*_g_), allowing diffusions and rearrangement of polymers.

Azobenzene, a well-known photoswitchable molecule, can undergo *trans*-*cis* photoisomerization upon UV irradiation.^24^ Applying azobenzene to polymer chains, versatile phenomena manipulated via light have been investigated, especially tuning their physicochemical properties.^25−27^ For instance, de Luna et al. have reported a supramolecular hydrogel based on azobenzene anions and cations with long alkyl chains, showing the healability after mechanical vibration.^28^ Meanwhile, Zhou et al. have presented a novel light-switchable adhesive with a tunable *T*_g_ of azobenzene-containing polymer upon UV irradiation.^29^

During the past decade, light-induced polymeric materials with healing abilities have been investigated for increasing their stability and lifetime. The healable fabrics, however, have been less developed due to the difficulty of maintaining the fiber structures. Herein, we design photohealable fabrics containing polystyrene (PS) and azobenzene-containing polymer (PAzo). With the structural support of the PS, the polymer blend fiber structures could be stabilized in the electrospinning process. The PS/PAzo blend fibers undergo photoinduced morphological changes while exposed to UV light, corresponding to the *trans*-*cis* photoisomerization and liquefaction of the PAzo sections with lower *T*_g_ (∼13.7 °C). Subsequently, the liquefied PAzo sections can be solidified using visible light to fix the modified fiber structures.

The liquefied areas of the PS/PAzo blend fibers with higher mobilities can also interact with other fibers with close contact. It should be noted that no additional material is required in the photoinduced self-healing of PS/PAzo blend fibers. After the fabrics are cut and overlapped in a T-shape with clamping, the damaged fabrics are shone with UV light, inducing the photoisomerization of the PAzo sections and causing the integration of the broken pieces. To our knowledge, this research marks the initial exploration of light-triggered self-healing fibers based on photoswitchable adhesives. The T-shape healing of the PS/PAzo blend fibers presents potential applications for next-generation smart clothing and fabric-based materials.

## Experimental Section

### Materials

Azobenzene-containing polymer (PAzo) with a weight-average molecular weight (*M*_w_) of 7 kg/mol was synthesized by reversible addition–fragmentation chain-transfer (RAFT) polymerization, as described in the Supporting Information. Polystyrene (PS, *M*_w_: 20 kg/mol) beads were obtained from Sigma-Aldrich. Toluene, ethanol (99.5%), and acetone were purchased from Echo Chemical. Dimethylformamide (DMF) was bought from Tedia. The electrospinning setup has been described in detail in our previous article.^30^

### Synthesis of Photoresponsive Azobenzene-Containing Monomers and Polymers

The azobenzene derivative (6-(4-(phenyldiazenyl)phenoxy)hexan-1-ol, AZOH) was synthesized by diazotization, which was based on previous studies with some modifications,^27^ as presented in Figures S1,2. Polymerizable methacrylate groups were functionalized on AZOH, as shown in Figures S3,4. The details of the synthesis process are shown in the Supporting Information.^31^

### Fabrication of PS Fibers and PS/PAzo Blend Fibers

Initially, polystyrene (PS) beads were accurately weighed and placed in a glass sample bottle, followed by adding an appropriate amount of DMF. The bottle is sealed tightly using parafilm to prevent evaporation. After stirring, the 20 wt % PS solution was prepared. For the PS/PAzo blend fibers, different ratios of PS/PAzo solutions (1:1, 4:1, 5:1, and 10:1) were also prepared.

The prepared solution was then transferred into a 5 mL polypropylene plastic syringe attached directly to a stainless steel capillary needle. After confirming that the polymer solution flowed smoothly from the needle, the syringe was secured in place on a microsyringe pump with a flow rate of 1.5 mL/h. The electrospinning setup utilized a horizontal frame, where the drum-type collector was parallel to the syringe at a rotational speed of 100 rpm. The distance was maintained at ∼13 cm, and a parchment paper covered the drum to prevent the adhesion of fibers.

The positive pole of the power supply was connected to the needle, while the drum collector was grounded. The voltage was then set to 15 kV, and a stable Taylor cone was formed at the tip of the needle, generating a jet stream. The electrospun fibers solidified upon landing on the rotating drum collector. After the electrospun fibers were collected, they were carefully removed from the drum and stored for subsequent use.

### Photoinduced Roughness of the PS/PAzo Blend Fibers

A piece of PS/PAzo blend fibers was first shone with a UV light source (∼365 nm, 136 mW cm^–2^) for 1 h, turning the PAzo section into *cis*-form. To prevent the decomposition of PS sections, the illumination of UV light should not be too long. Subsequently, to change the *cis*-form back to *trans*-form for solidification, the PS/PAzo blend fibers were placed under visible light (∼520 nm, 370 mW cm^–2^) for another 20 min. After the light-induced process, the water contact angles (WCA) of the PS/PAzo blend fibers were tested to confirm the hydrophilicity.

### Self-Healing Experiments of the PS/PAzo Blend Fibers

The photoinduced self-healing process capitalized on the light-induced solid–liquid phase transition property inherent in PAzo sections. When the PS/PAzo blend fibers were exposed to UV light, the PAzo sections were transformed into a *cis*-state, indicating a liquid state of PAzo. Meanwhile, two PS/PAzo blend fiber pieces were overlapped. After shining with UV light (136 mW cm–2, wavelength ∼365 nm) for 20 min, the overlapped PS/PAzo blend fabrics were subjected to pressure and maintained for 24 h. The liquid-state PAzo gradually flowed and made contact with each other. Subsequently, a visible light was employed to convert the liquid PAzo back into the solid state, thus completing the self-healing process.

### Structure Characterizations and Analyses

A scanning electron microscope (SEM, JEOL JSM-7401F) was applied at an acceleration voltage of 5 kV to identify the surface morphologies of the fibers. The fibers were covered with a 4 nm layer of platinum before conducting the SEM measurements. To stabilize the fiber structures, the samples were exposed to visible light for 20 min before each SEM measurement. Time-evolved UV–vis absorption and reflection spectra were tested by an ultraviolet–visible spectrometer (Hitachi U4100) to record the photoisomerization of the azobenzene polymers. Water contact angles (WCA) were confirmed by a goniometer (FTA 125, First Ten Ångstroms) to present the hydrophobicity of the surface of the fibers. WCA measurements with dropping water droplets (4 μL) under ambient conditions were obtained in a static state.

## Results and Discussion

**Figure 1:**
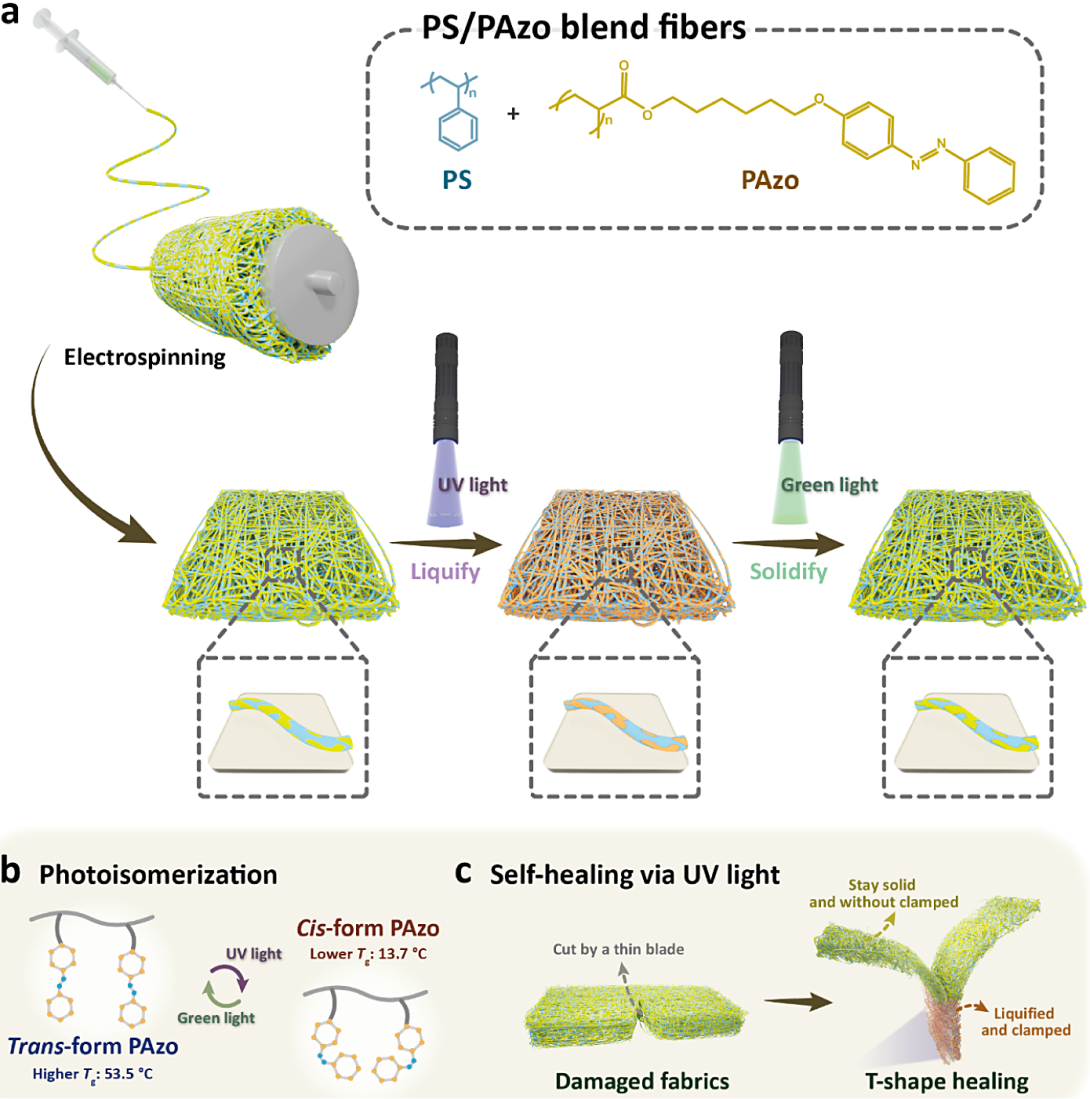
Conceptual illustration and self-healing process of the PS/PAzo blend fibers. (a) Electrospinning process of PS/PAzo blend fibers with photoinduced roughness. (b) Photoisomerization of PAzo and related *T*_g_ changes via UV and visible lights. (c) Graphical illustrations of the self-healing process of the PS/PAzo blend fibers via UV light.

The PS/PAzo blend fibers comprise polystyrene (PS) and azobenzene-containing polymer (PAzo), as illustrated in Figure 1a. Notably, due to the relatively low molecular weight (*M*_W_) of PAzo (∼10000 g/mol), the mechanical strength is insufficient to form stable fiber structures independently. To address this issue, we apply PS as a supporting material, which is crucial for forming stable fiber structures. In the electrospinning process, a blended polymer solution (PS:PAzo = 5:1, in DMF) is mixed and spun at a selected voltage (15 kV). In the fabrication of the PS/PAzo blend fabrics, PS is selected to provide support and maintain the fiber structures because PS does not deform upon UV irradiation. In addition, blending PS enhances the mechanical properties of the fabrics. Therefore, PS does not play a significant role in supporting the healing ability. Instead, the PAzo sections are mainly responsible for the healing ability by undergoing *trans*-*cis* isomerization, allowing them to attach to other fibers upon appropriate adhesion. After the fabrication of the PS/PAzo blend fibers, the samples are exposed to UV light to induce photoinduced morphological changes corresponding to the liquefaction of the PAzo sections. The liquefied PAzo sections are subsequently solidified using visible light to stabilize the modified fiber structures.

Looking deeply into the mechanism behind the morphological changes in the PS/PAzo blend fibers reveals the crucial role of the photoisomerization of the azobenzene groups in the polymer, as depicted in Figure 1b. Upon UV exposure, the azobenzene groups in the PAzo sections undergo *trans*-to-*cis* photoisomerization, leading to configurational differences in the polymer chains and inducing transitions from a solid state to a liquid state by lowering the *T*_g_ values of the PAzo below room temperature (∼13.7 °C). The azobenzene moieties in the microphase-separated polymer blends are selectively fluidized, increasing the mobilities of the PAzo sections and causing molecular rearrangement. Subsequent exposure to visible light triggers the *cis*-to-*trans* photoisomerization of the azobenzene groups in the PAzo sections, solidifying the polymers by raising the *T*_g_ of the PAzo back above room temperature (∼53.5 °C). Finally, the surface morphologies of the PS/PAzo blend fibers are fixed.

Interestingly, the liquefied areas of the PS/PAzo blend fibers with higher mobilities not only influence the surface roughness of the single fiber but also interact with other fibers as long as the compact contact area is sufficient. As shown in Figure 1c, in addition to the surface roughness induced by UV irradiation, the photoinduced self-healing properties are also demonstrated. Initially, the fabrics are cut with a thin blade. The damaged (cut) fabrics are then overlapped in a T-shape and clamped with a commercial clip for a while. When pressure is applied to the PS/PAzo blend fabrics, the contact area increases. Subsequently, UV light is turned on to generate the photoisomerization of the PAzo, initiating the healing process. The T-shape healing of the PS/PAzo blend fibers is demonstrated, showcasing potential applications for smart clothing and fabric-based materials.

**Figure 2:**
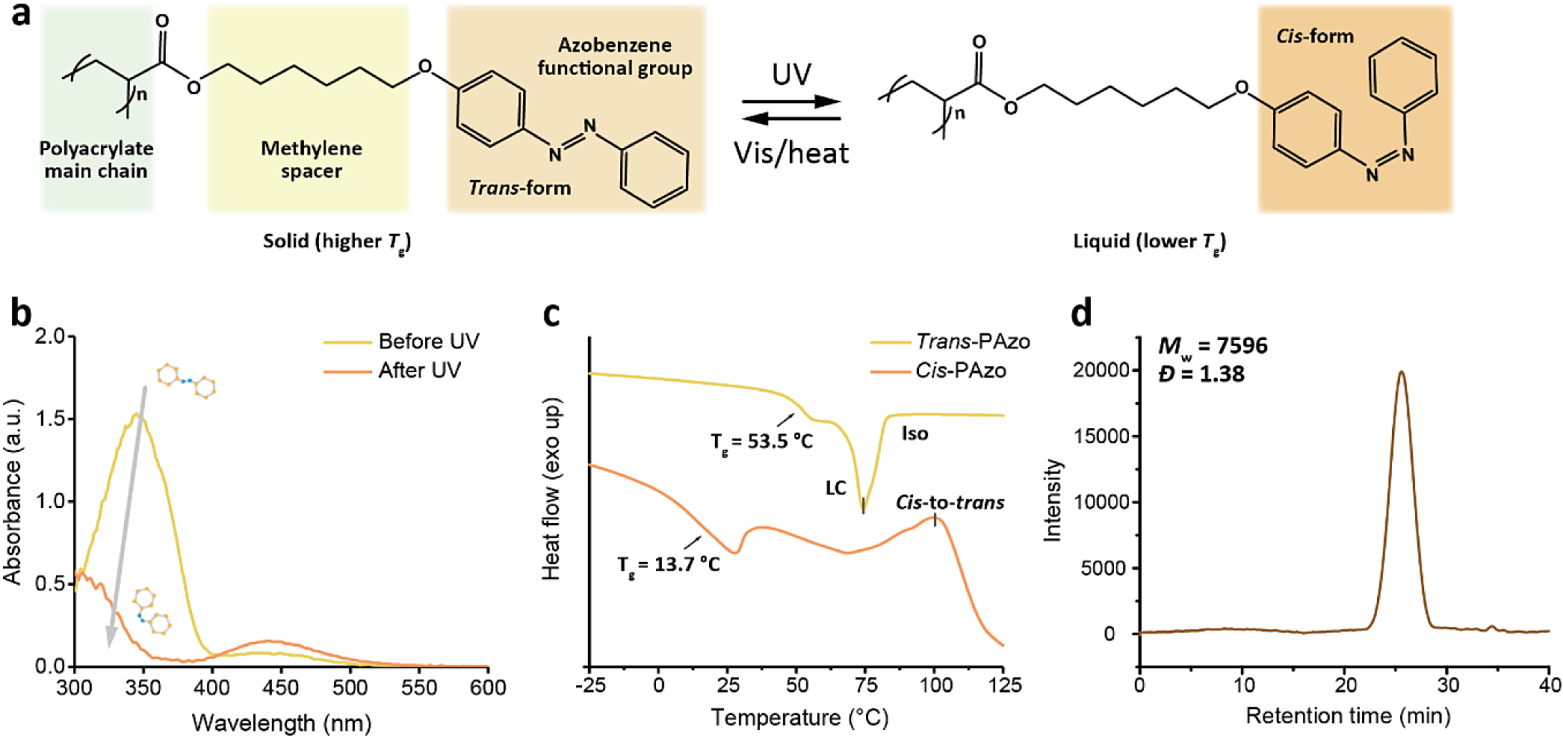
Photoswitching of the PAzo sections. (a) Reversible photoisomerization mechanism of the PAzo. (b) UV–vis absorption spectra of the PAzo before and after UV irradiations. (c) DSC curves of the *trans*- and *cis*-PAzo. *T*_g_: glass transition temperature; LC, liquid crystalline phase; Iso, isotropic phase. (d) GPC data of PAzo with a *M*_w_ of 7 kg/mol.

The PAzo sections of the PS/PAzo blend fibers are synthesized through reversible addition–fragmentation chain-transfer (RAFT) polymerization, as displayed in Figure S3. According to previous studies,^32,33^ the PAzo exhibits reversible photoisomerization during UV and visible light irradiation, as depicted in Figure 2a. The PAzo structures can be divided into three key components: the polyacrylate main chain, the methylene spacer, and the azobenzene functional group. The polyacrylate main chain serves as the polymer backbone, while the length of the flexible methylene spacer influences the glass transition temperature (*T*_g_) of the PAzo. The azobenzene functional groups, the vital parts of the polymer side chain, can undergo reversible *trans*-*cis* isomerization. Initially, the *trans*-state PAzo presents a solid state with a *T*_g_ above room temperature (∼53.5 °C) before UV light exposure. After UV illumination, the azobenzene groups undergo *trans*-to-*cis* isomerization, forming a liquid *cis*-state PAzo with a *T*_g_ below room temperature (∼13.7 °C). The *cis*-state PAzo can also be reverted to the *trans*-state using visible light or heat. The storage moduli (*G*′) and loss moduli (*G*′’) of the PAzo polymer film in amplitude sweep are displayed in Figure S5. The time-dependent curves of the loss modulus (*G*′’) and storage modulus (*G*′) of the PAzo polymer film before and after UV irradiation are presented in Figure S6. After UV irradiation, the PAzo polymer film presents decreased and unstable *G*′ and *G*′’ because of the lower *T*_g_ of *cis*-PAzo (∼13.7 °C) below room temperature, indicating a much softer and “liquid-like” structures.

The reversible *trans*-*cis* photoisomerization behavior of the PAzo solutions can also be tracked by UV–vis absorption spectra, as presented in Figure 2b. Initially, the PAzo in solutions, predominantly in the *trans* states, exhibits a robust π–π* absorption band in the UV range and a weak *n*-π* absorption band in the visible range before UV exposure. After UV irradiation, the peak of the π–π* absorption band for the *trans* isomer decreases. In contrast, the peak of the *n*-π* absorption band for the *cis*-isomer increases, indicating a rise in the *cis*-PAzo ratio. Figure 2c shows the thermal behavior of the photoswitchable PAzo analyzed by differential scanning calorimetry (DSC). The *trans*-PAzo, possessing a *T*_g_ of ∼53.5 °C, exhibits a phase transition at ∼74 °C, attributed to π–π stacking in its solid state. In contrast, the *cis*-PAzo, with a *T*_g_ of ∼13.7 °C, is lower than the room temperature. Furthermore, a broad exothermic band is observed at ∼98 °C for the *cis*-PAzo because of the thermal *cis*-*trans* isomerization. The storage moduli (*G*′) and loss moduli (*G*′’) of the PS/PAzo blend polymer films in amplitude sweep are displayed in Figure S7. The temperature-dependent and loss factor (tan δ) curves of loss modulus (*G*′’) and storage modulus (*G*′) of the PS/PAzo blend polymer films before and after UV irradiation are presented in Figure S8. The rheology data agree well with the DSC data, indicating the *T*_g_ of the polymers. Figure 2d presents the gel permeation chromatography (GPC) analysis of PAzo. The molecular weight (*M*_W_) and polymer dispersity index (PDI) of PAzo are calculated to be 7569 g/mol and 1.38, respectively.

**Figure 3:**
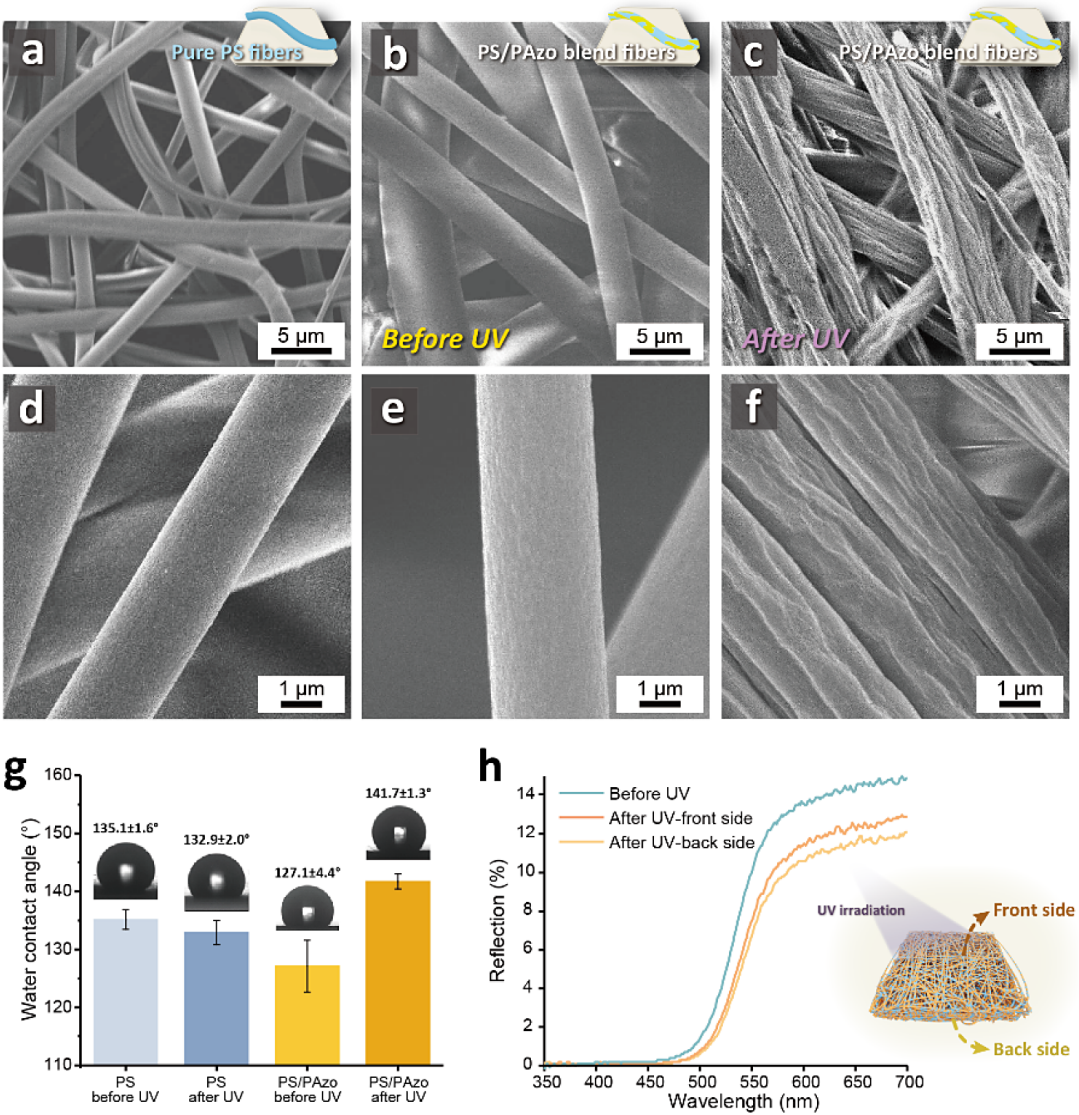
Structural characterizations of the PS/PAzo blend fibers. (a–f) Top-view SEM images: (a,d) pure PS fibers; (b,e) PS/PAzo blend fibers before UV irradiation; and (c,f) PS/PAzo blend fibers after UV irradiation. (g) Water contact angles of the pure PS fibers and PS/PAzo blend fibers before and after UV irradiations. (h) Reflectance spectra of the PS/PAzo blend fibers before and after UV irradiations, including the front and back sides.

Upon UV irradiation, the PS/PAzo blend fibers present a surface roughness change while the photoisomerization of PAzo occurs, as shown in Figure 3a–f. Compared with the pure PS fibers (Figure 3a,d), the surfaces of the PS/PAzo blend fibers are coarse-grained structures caused by the higher polymer concentration after adding PAzo in the electrospinning process, as displayed in Figure 3b,e. Additional SEM images of the pure PS fibers at different magnifications are displayed in Figure S9. Interestingly, the PS/PAzo blend fibers (PS:PAzo = 1:1, in DMF) generate several furrows and wrinkles on the surfaces upon UV irradiation, as shown in Figure 3c,f. It is worth noting that, due to the lower *M*_w_ of the PAzo, the mechanical properties are inferior, making them unsuitable for standalone fiber preparation. With the support of the PS fibers as a backbone material, the mechanical strength of the PS/PAzo blend fibers enables the maintenance of the fiber structures.

Figure 3g displays the water contact angles and the hydrophobicity of the PS fibers and PS/PAzo blend fibers. Initially, PS and PAzo are hydrophobic materials, while the three-dimensional structures formed by electrospinning enhance their hydrophobicity. After being exposed to light, the contact angles of the PS/PAzo blend fibers increase from ∼127.1 to ∼141.7°, while the contact angles of the PS fibers are of similar value (∼135°), providing macroscopic evidence of the morphological changes that create hierarchical structures resembling those of a lotus leaf.

Figure 3h depicts a comparison of the PS/PAzo blend fibers with a blending ratio of 5:1 before and after light exposure and on the front and back sides. The reflection intensity of the fibers is higher before light exposure (blue line). After exposure to light, the intensity slightly decreases and shifts toward the red spectrum (orange line), aligning with the phenomenon observed by the naked eye, transitioning from yellow (*trans*-PAzo) to orange (*cis*-PAzo). As UV light cannot fully penetrate the fibers, the measured reflection spectrum at the back side of the fibers falls between pre-exposure and postexposure intensities.

**Figure 4:**
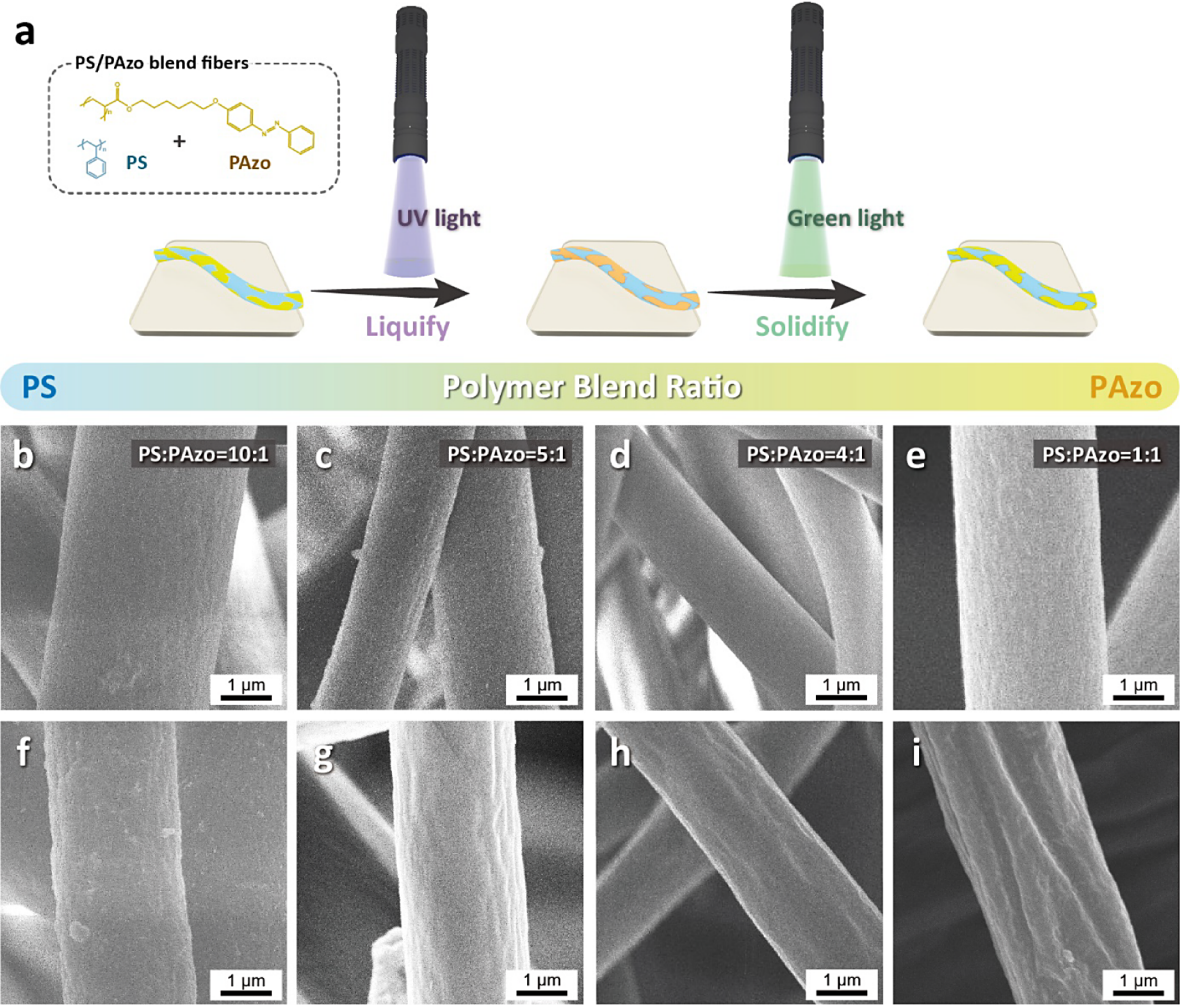
PS/PAzo blend fibers with different polymer blend ratios. (a) Schematic illustration of the PS/PAzo blend fibers by shining with UV and visible lights to liquify and solidify the PAzo sections in the fibers. (b–e) Top-view SEM images of the PS/PAzo blend fibers with different PS/PAzo blend ratios before UV irradiations: (b) 10:1, (c) 5:1, (d) 4:1, and (e) 1:1. (f–i) Top-view SEM images of the PS/PAzo blend fibers with different PS/PAzo blend ratios after UV irradiations: (f) 10:1, (g) 5:1, (h) 4:1, and (i) 1:1.

Figure 4 illustrates the morphological variations of the fibers under UV light exposure at different PS/PAzo blending ratios. The SEM measurements of the PS/PAzo blend fibers are taken before and after UV irradiation. The fibers are shone with visible light after UV irradiation to stabilize the wrinkled surfaces, as shown in Figure 4a.

The SEM images reveal that, regardless of the blending ratio, the surfaces of the PS/PAzo blend fibers become rougher, as displayed in Figure 4b–i. Before UV irradiation (Figure 4b–e), the fibers present relatively smooth surfaces, in contrast to those that shone with UV light (Figure 4f–i). Meanwhile, it can be seen that the surface roughness of the fibers increases significantly with the percentage of PAzo, resulting in wrinkled structures. The results indicate that more PAzo chains in the fibers can cause more photoisomerization to transform into a relatively mobile liquid state. Figure 4e,i, where the PS/PAzo blending ratio is 1:1, shows the most significant morphological changes after UV exposure. The result can be attributed to the photosensitivity of the PAzo, which transitions from a solid state to a liquid state under UV light irradiation, resulting in wrinkled patterns resembling water ripples on the fiber surfaces. The surface morphologies of the PS/PAzo blend fabrics with other blend ratios are also presented in Figure S10.

**Figure 5:**
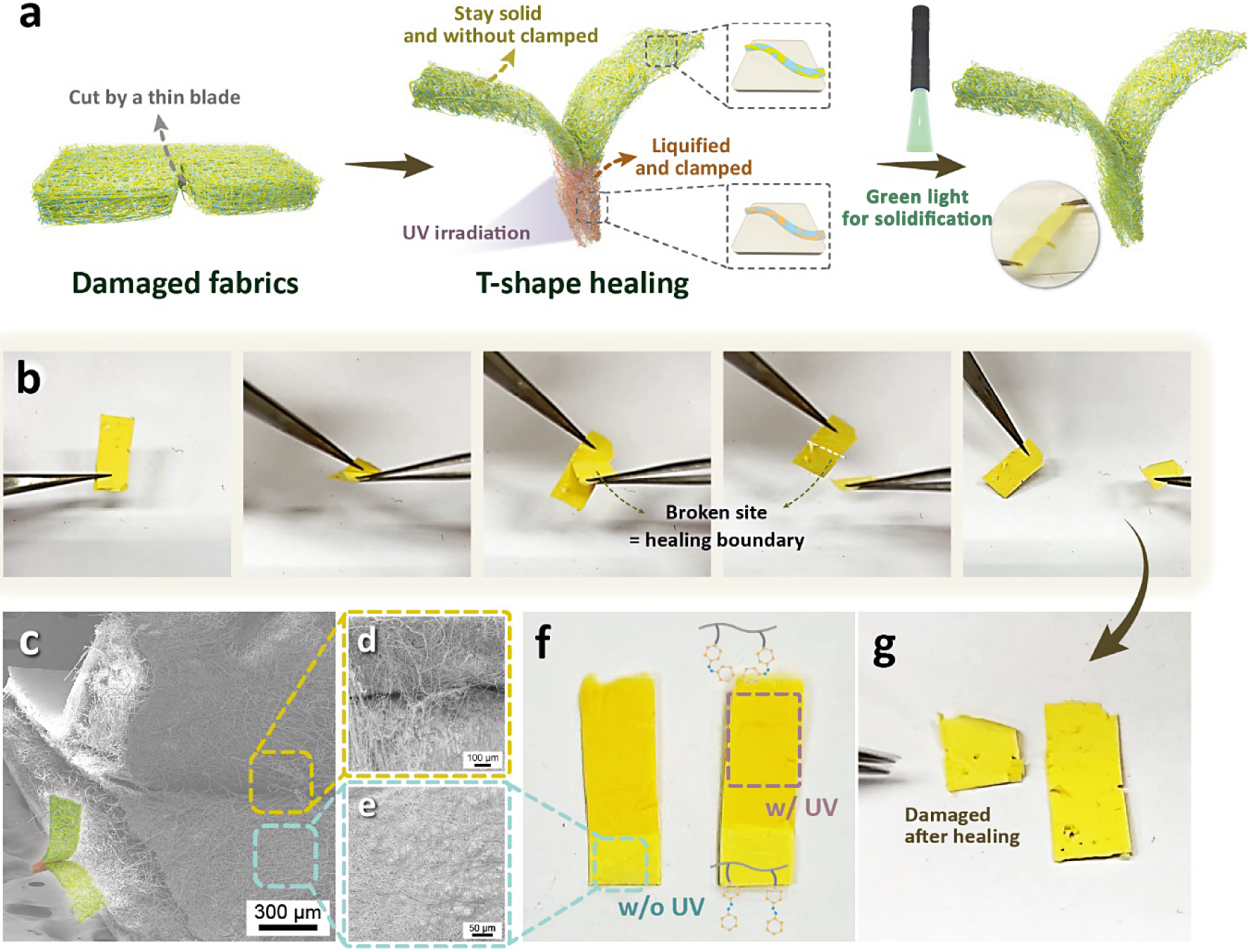
Photoinduced self-healing process of the PS/PAzo blend fibers. (a) Illustrations of the damaged fabrics and the T-shape healing process. (b) Photographs of the self-healed fabrics and redamaged fabrics, where the broken boundaries of the fabrics can be seen. (c–e) SEM images of the self-healed fabrics: (c) the self-healed boundary of the T-shape structures, (d) the liquified and clamped locations of the fabrics, and (e) the location without UV irradiation and clamped. (f,g) Photographs of the pristine and damaged PS/PAzo blend fibers after the self-healing process: (f) two pieces of the fabrics with and without UV irradiation and (g) the damaged self-healed fabrics after the healing process. The broken self-healed boundary is irregular.

Based on the surface morphology changes in the PS/PAzo blend fibers, we further demonstrate the photoinduced self-healing via UV irradiation. Figure 5a illustrates the damaged fabrics and the T-shape healing process. A piece of fabric is cut by a thin blade. Subsequently, two ends of the damaged fabrics are placed in a T-shape to heal the fabrics. The overlapped regions are sandwitched between glass substrates and clamped with commercial clips to maintain contact of the fabrics. The overlapped regions of the T-shape fabrics are then shone with UV light to initiate the photoisomerization of the PAzo section. After shining with UV light for 20 min and subjected to pressure for 24 h, the fabrics are healed. A green light solidifies the liquid PAzo, completing the repairing function, as shown in the photograph in Figure 5a. Figure 5b shows the separation of two repaired PS/PAzo blend fibers, while Figure 5g presents the results after separation. The broken sites in the separation of the repaired PS/PAzo blend fibers are also the photohealing boundaries of the fabrics, which makes separation much more difficult, resulting in irregularly broken fabrics. In addition to T-shape healing, we also conduct overlapped healing in selective regions by pressing for 24 h after UV irradiation for 20 min, as shown in Figure S11. With both T-shaped and overlapped photoinduced self-healing, the fabrics present much better healing ability compared with fabrics without UV irradiation.

From the SEM images in Figure 5c–e, some fiber fractures can be observed. The repair process leverages the photoinduced solid–liquid phase transition characteristics of the azobenzene molecules. When the repair fibers are exposed to UV light, the transformation of PAzo into a liquid *cis*-state is induced. In this state, the liquid PAzo slowly flows and comes into contact with each other (Figure 5d), showing the different morphologies compared with the fabrics without being clamped and UV irradiation (Figure 5e). Enlarged SEM images, indicating the boundary of two pieces and the regions of fibers sticking together, are shown in Figure S12. Additional SEM measurements of the PS fabrics before and after UV irradiation for 24 h are conducted, as displayed in Figure S13. After UV irradiation, the fiber structures are still maintained, although the *M*_W_ might be decreased due to chain scission, as reported in previous studies.^34,35^ The difference between the self-healed regions and the other regions is also distinguished in their actual appearance, as shown in Figure 5f. Once two fibers are bonded together like a glue, attempting to separate them would disrupt the fiber structures (Figure 5g). Tensile tests have been conducted to confirm the changes in mechanical properties before and after the healing process, as displayed in Figure S14. Higher maximum forces are observed after the overlapped healing process, attributed to both the photoinduced self-healing and the double-layer structures in the overlapped regions.

## Conclusions

In this study, we have successfully developed a novel photohealable fabric, addressing the vulnerability of polymer fabrics to mechanical damage. Our approach utilizes a blend of polystyrene (PS) and azobenzene-containing polymer (PAzo) as the fiber material. This unique combination allows the PAzo sections within the PS/PAzo blend fibers to undergo reversible *trans*-*cis* photoisomerization upon UV light exposure. This transition transforms the material from a solid to a liquid state, facilitated by the notably low glass transition temperature (*T*_g_) of about 13.7 °C, making the material amenable to reshaping and repair. The subsequent application of visible light solidifies the modified fiber structures, stabilizing them postmodification. The innovative aspect of our research lies in the photoinduced self-healing process, capitalizing on the mobility of the liquefied areas of the PS/PAzo blend fibers. This process, triggered by UV light and followed by visible light treatment, has demonstrated effective healing of damaged fabrics. The successful T-shape healing of the PS/PAzo blend fibers not only underscores the practicality of this approach but also opens up new avenues for the application of these fibers in next-generation smart clothing and fabric-based materials. In the future, we will investigate the photoinduced self-healing ability by coating the PAzo on commercial fabrics, paving the way for more sustainable and durable textile products.

## Supporting Information Available

Synthetic scheme and ^1^H NMR spectra of azobenzene derivative and azobenzene-containing polymer; rheology data of PAzo polymer films and PS/PAzo blend polymer films in different times and temperatures; SEM images of the pure PS fibers before and after exposed to UV light for 24 h, the PS/PAzo blend fibers with other polymer blend ratios, and the self-healed fabrics; tensile tests of pristine and overlapped healing fabrics; photos of the overlapped and T-shape photoinduced self-healing of the PS/PAzo blend fabrics (PDF)
