# Supplementary material for: Photo-Healable Fabrics: Achieving Structural Control via Photochemical Solid–Liquid Transitions of Polystyrene/Azobenzene-Containing Polymer Blends
Source: ACS Appl Mater Interfaces. 2024 May 21;16(22):29153–61. doi: 10.1021/acsami.4c02578 (PMC11163394; doi:10.1021/acsami.4c02578)
Supplement: Supplementary file 1 — am4c02578_si_001.pdf [file am4c02578_si_001.pdf]

Supporting Information

Photo-Healable Fabrics: Achieving Structural Control via Photochemical Solid–Liquid Transitions of Polystyrene/Azobenzene-Containing Polymer Blends

Yi-Fan Chen,[1,†] Meng-Ru Huang,[1,†] Yen-Shen Hsu,[1] Ming-Hsuan Chang,[1] Tse-Yu Lo,[1] Bhaskarchand Gautam,[1] Hsun-Hao Hsu,[1] and Jiun-Tai Chen[1,2*]

[1]Department of Applied Chemistry, National Yang Ming Chiao Tung University, 300093 Hsinchu, Taiwan

[2]Center for Emergent Functional Matter Science, National Yang Ming Chiao Tung University, 300093 Hsinchu, Taiwan

*To whom correspondence should be addressed. E-mail: jtchen@nycu.edu.tw.

†Yi-Fan Chen and Meng-Ru Huang contributed equally to this work.

Synthesis of Azobenzene Derivative (AZOH)

7.29 mL of aniline was dissolved in 20 mL of 37 wt % hydrochloric acid in an ice bath. Then, 6.1 g of sodium nitrite ($NaNO_2$) was also dissolved in 25 mL of DI water. The cooled $NaNO_2$ solution was dripped into the aniline/hydrochloric acid solution, forming the diazotizing salt. A solution of 11.28 g of phenol in 50 mL of 10 wt % sodium hydroxide solution was prepared and slowly dripped into the diazotizing salt-containing solution while continuously stirring, forming a yellow precipitate. The pH value of the solution was adjusted by 1 M HCl and NaOH solution. After filtration, the yellow precipitate was washed with deionized water. The crude product was dissolved in methanol and dripped into ice DI water for further purification. The yield of 4-phenylazophenol was approximately 89%.

14.49 g of 4-phenylazophenol, 10.1 g of potassium carbonate (K_2CO_3), and 0.03 g of potassium iodide (KI) were dissolved in 80 mL of dimethylformamide (DMF). 10.57 mL of 6-chloro-1-hexanol was added into the solution and refluxed at 110 °C for 24 h. After cooling the reflux reaction to room temperature, the upper layer was extracted and added dropwise to excess ice DI water. A yellow precipitate was collected by vacuum filtration and dried. The crude product was recrystallized using methanol, and the yield was approximately 75%.

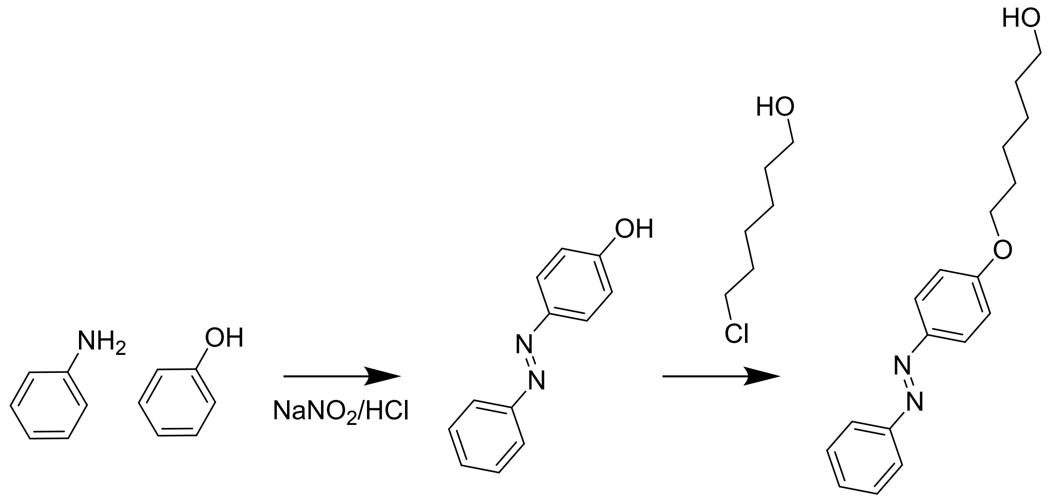

Figure S1. Synthetic scheme of the azobenzene derivative (AZOH).

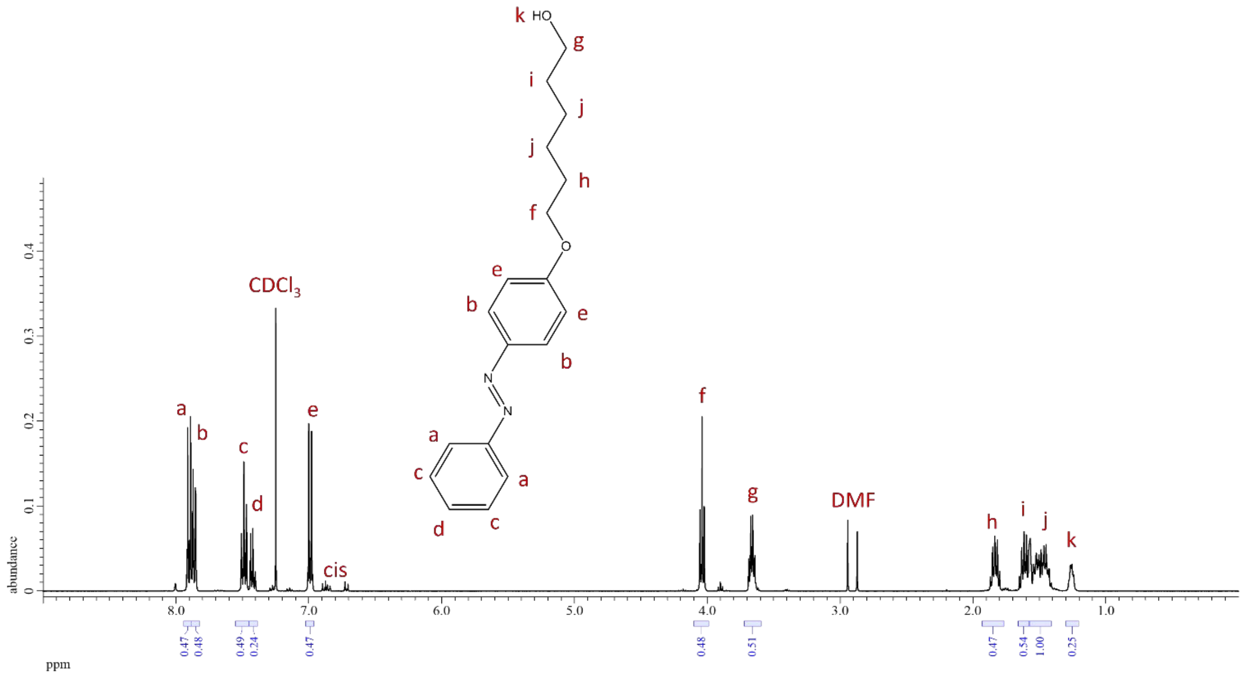

Figure S2. ^{1}H NMR spectrum of AZOH.

Polymerization of Azobenzene-Containing Polymer (PAzo)

16.36 g of 6-(4-(phenyldiazenyl)phenoxy)hexan-1-ol (Azo2) was dissolved in 7.64 mL of triethylamine and 50 mL of dichloromethane (DCM). The solution was placed in an ice bath, and 6.95 mL of acryloyl chloride was slowly added. After completing the addition of acryloyl chloride, the mixture was stirred at room temperature for 20 h. The dichloromethane solvent was removed using a rotary evaporator. The obtained solid was dissolved in ethyl acetate, and the pH value was confirmed at ~7. The solution was extracted with DI water, and the organic layer was evaporated using a rotary evaporator. The yield was 71%.

Azobenzene polymer (PAzo) was synthesized through free-radical polymerization (FRP). Firstly, the anisole was degased by introducing nitrogen for 4 h. 1.5 g of 6-(4-(phenyldiazenyl)phenoxy)hexyl methacrylate and 30 mg of initiator 2,2'-azobisisobutyronitrile (AIBN) were dissolved in 4 mL of anisole. The solution was placed in an oil bath and heated to 80 °C, followed by stirring for 24 h. After cooling to room temperature, the solid was precipitated by slowly adding excess methanol dropwise. The precipitate was collected using a centrifuge and dissolved in tetrahydrofuran (THF). The solution was reprecipitated by dripping into methanol, repeating until the methanol was clear. The yield of the azobenzene polymer was approximately 49.8%, with a PDI of 1.38 and a M_w of 7596 g/mol.

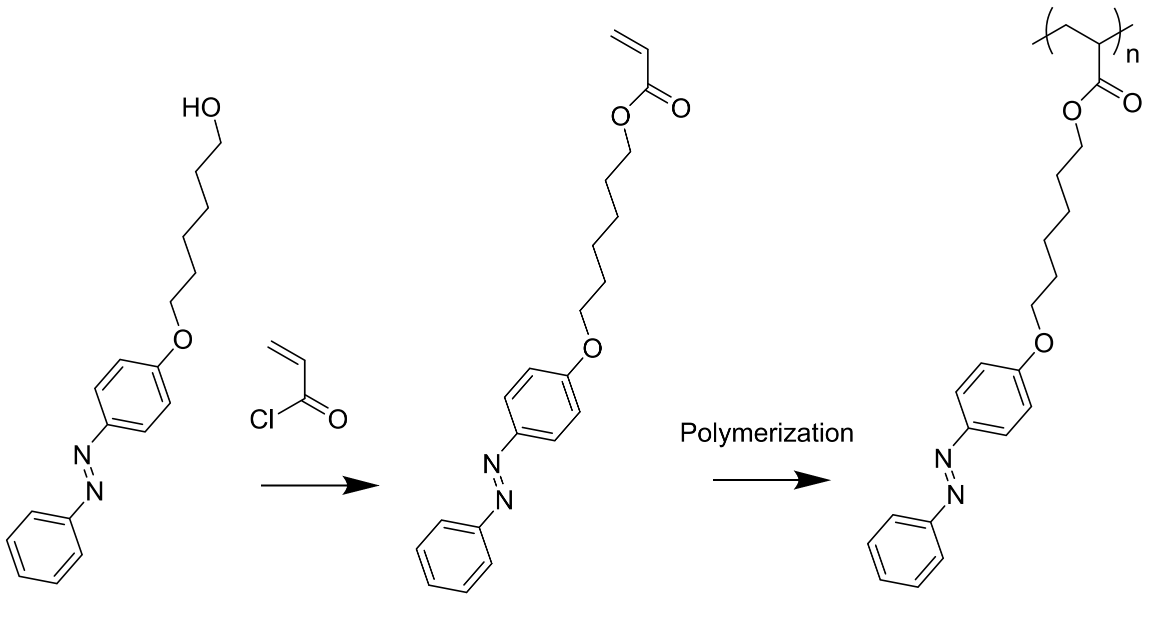

Figure S3. Synthetic scheme of the azobenzene-containing polymer (PAzo).

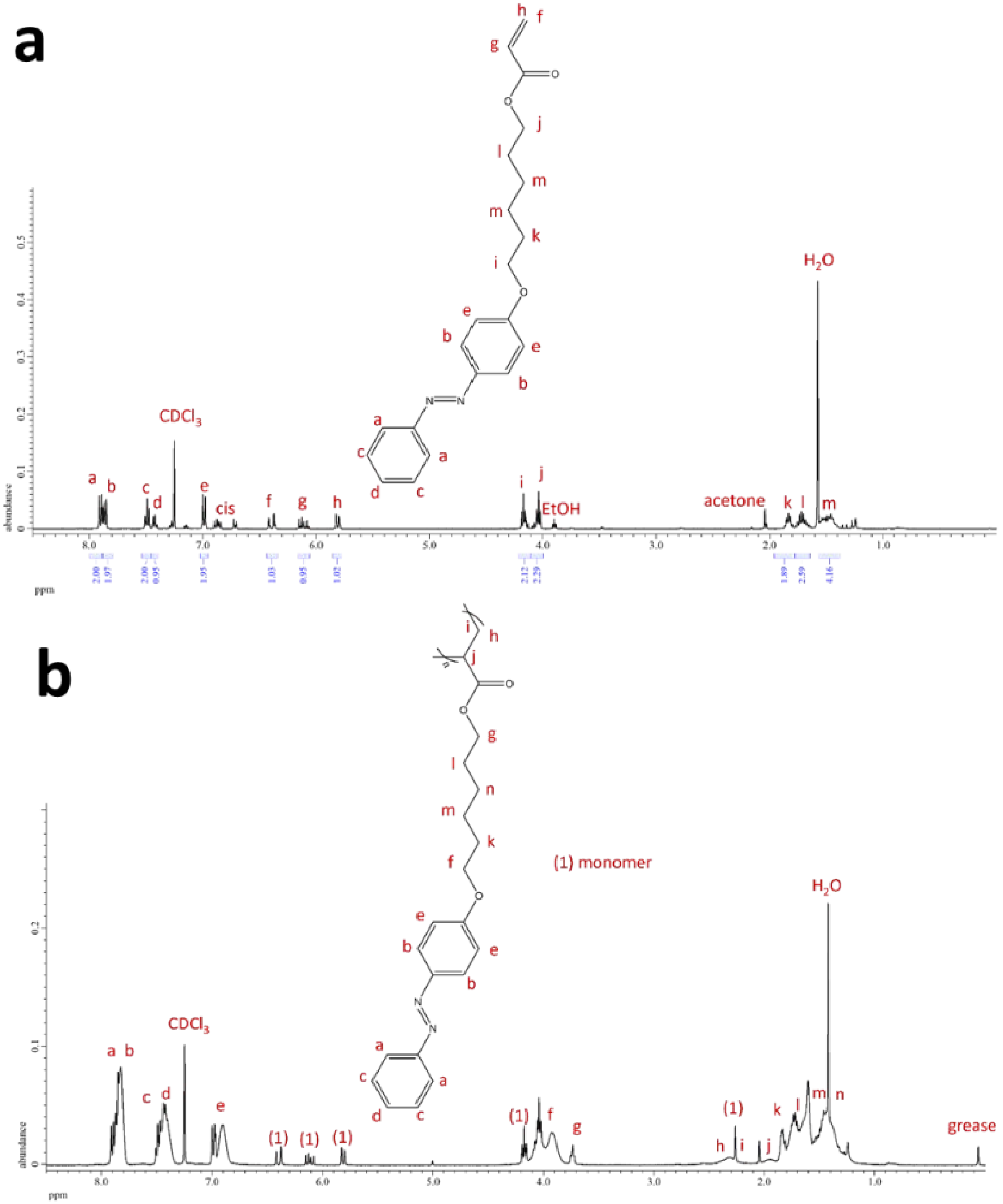

Figure S4. ^{1}H NMR spectra of the azobenzene-containing (a) monomer and (b) polymer (PAzo).

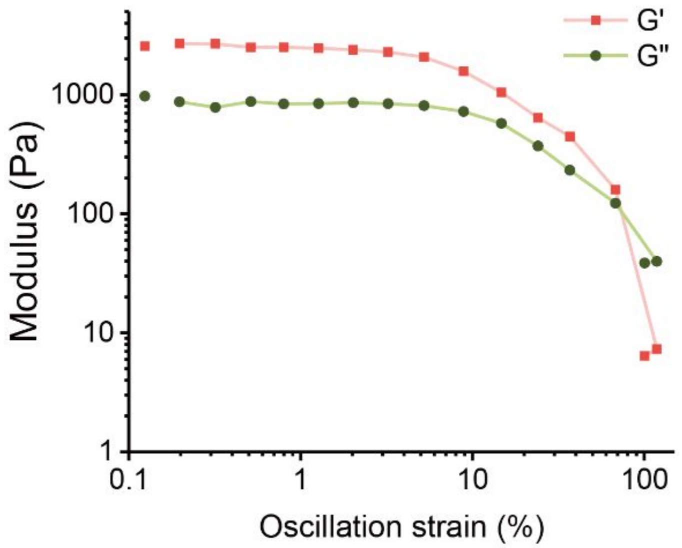

Figure S5. Rheology data of the PAzo polymer film displaying storage moduli (G') and loss moduli (G'') in amplitude sweep (ω = 10 rad/s, 30 °C).

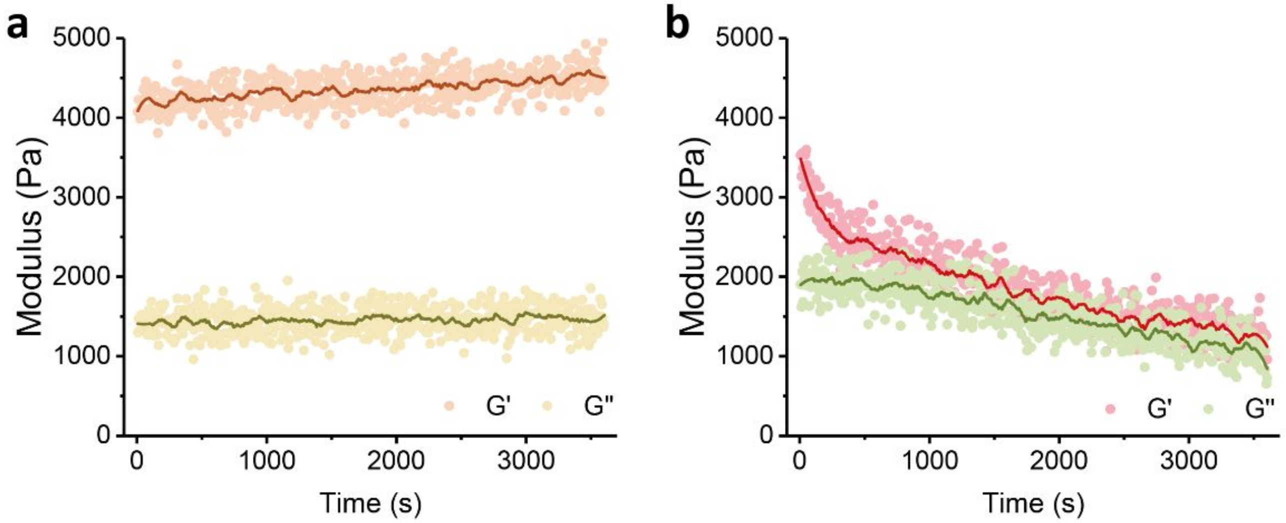

Figure S6. Rheology data of the PAzo polymer film displaying storage moduli (G') and loss moduli (G'') in different times (ω = 10 rad/s, 30 °C). Modulus curves of the PAzo polymer film (a) before UV irradiation and (b) s after UV irradiation.

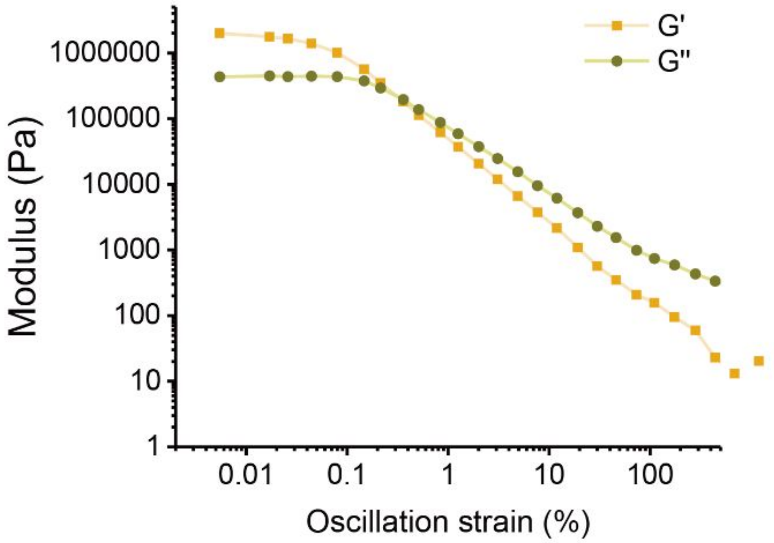

Figure S7. Rheology data of the PS/PAzo blend polymer films displaying storage moduli (G') and loss moduli (G'') in amplitude sweep (ω = 10 rad/s, 30 °C).

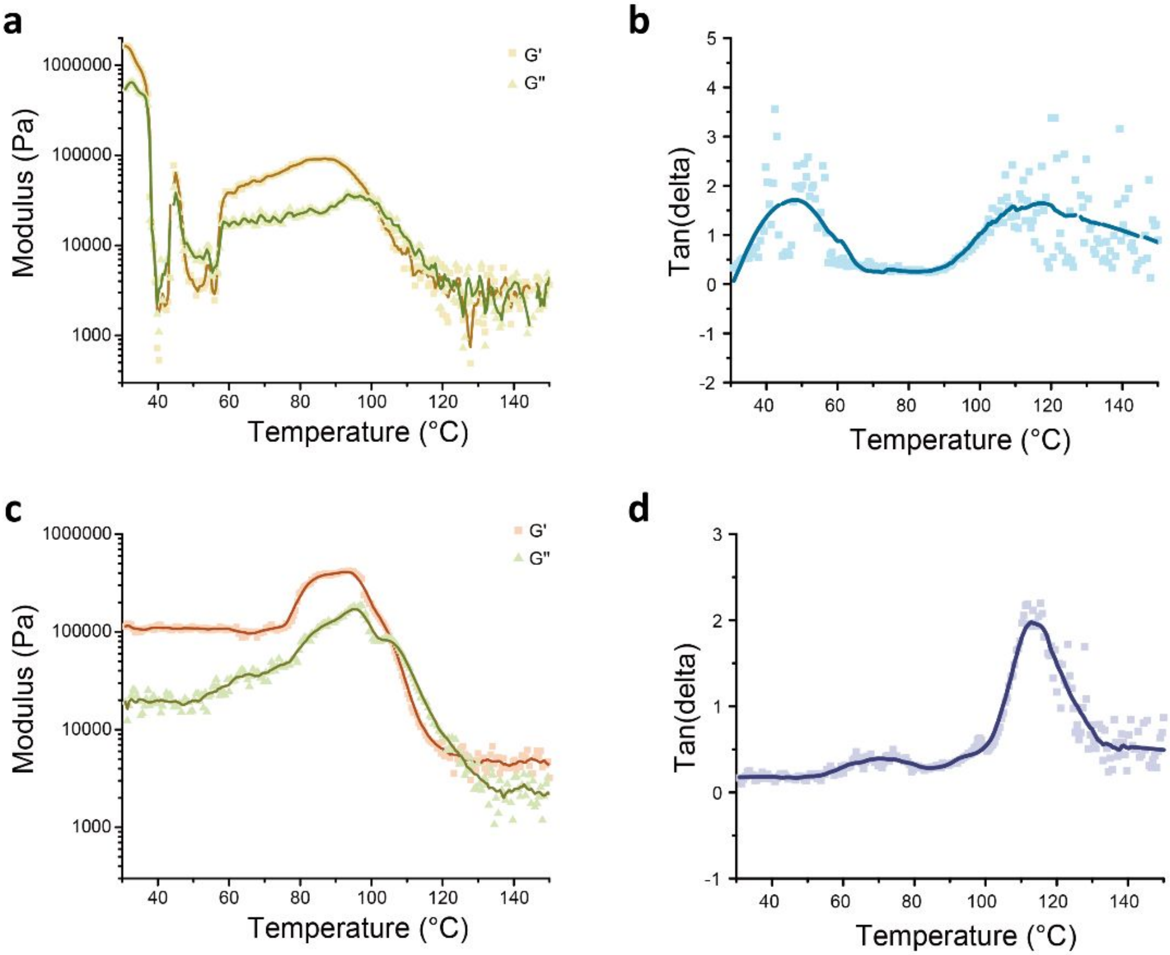

Figure S8. Rheology data of the PS/PAzo blend polymer films displaying storage moduli (G') and loss moduli (G'') in different temperatures (ω = 10 rad/s, 30 °C). (a) Modulus and (b) loss factor (tan δ) curves of the PS/*trans*-PAzo blend polymer films before UV irradiation. (c) Modulus and (d) loss factor (tan δ) curves of the PS/*cis*-PAzo blend polymer films after UV irradiation.

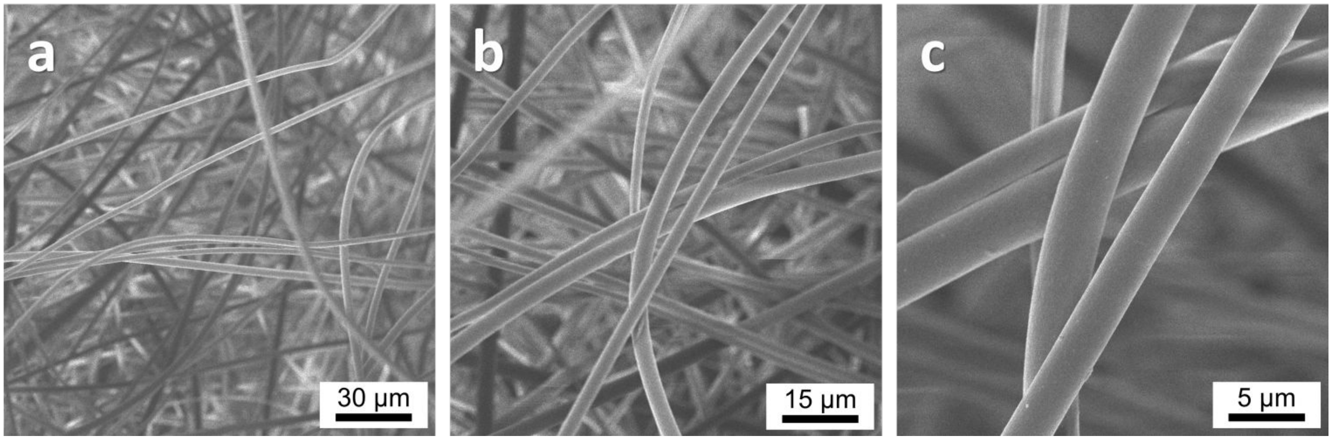

Figure S9. SEM images of pure PS fibers at (a) 500x, (b) 1000x, and (c) 3000x magnifications.

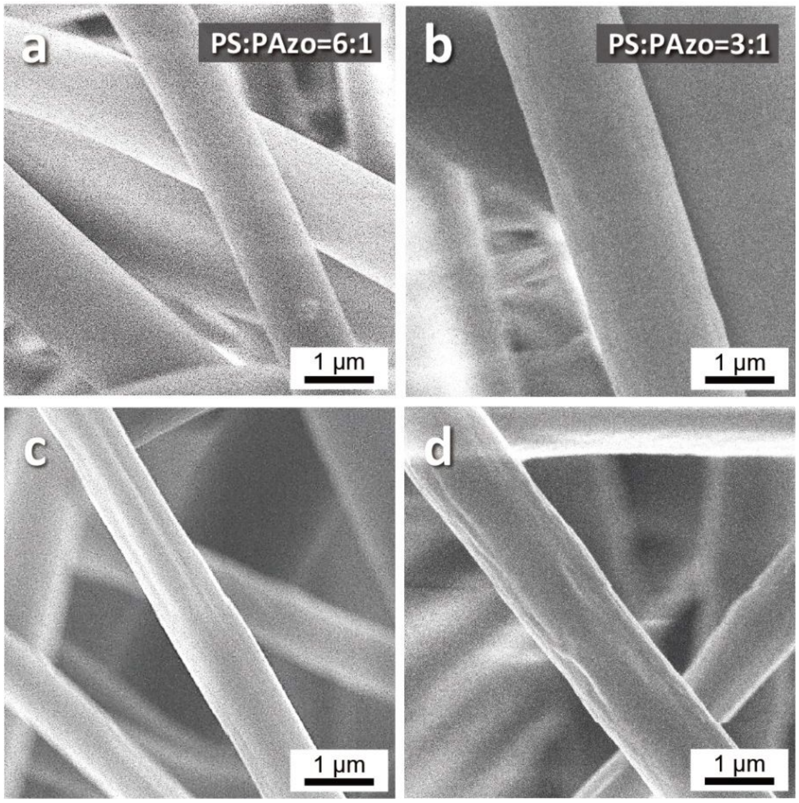

Figure S10. PS/PAzo blend fibers with other polymer blend ratios. (a,b) Top-view SEM images of the PS/PAzo blend fibers with different PS/PAzo blend ratios before UV irradiations: (a) 6:1 and (b) 3:1. (c,d) Top-view SEM images of the PS/PAzo blend fibers with different PS/PAzo blend ratios after UV irradiations: (c) 6:1 and (d) 3:1.

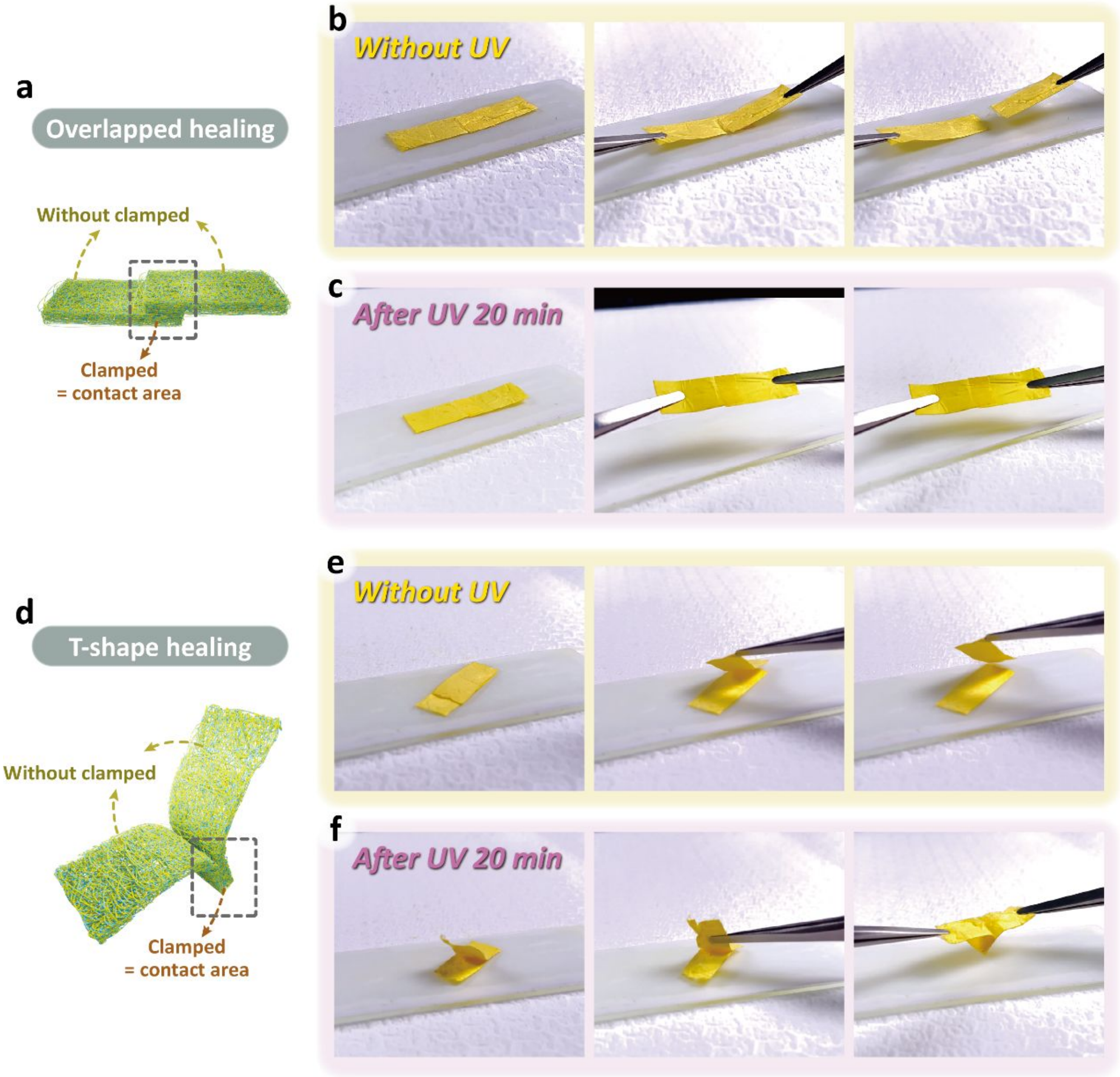

Figure S11. Overlapped and T-shape photoinduced self-healing of PS/PAzo blend fabrics. (a) Scheme of the overlapped structure. Overlapped fabrics (b) without UV irradiation and (c) under UV irradiation for 20 min. (d) Scheme of the T-shaped structure. T-shaped fabrics (e) without UV irradiation and (f) under UV irradiation for 20 min.

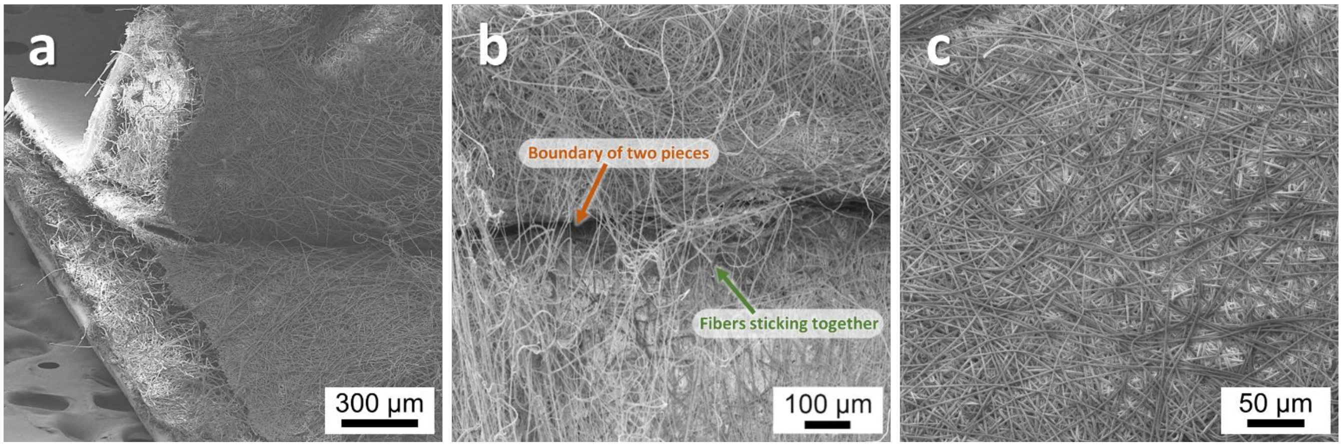

Figure S12. SEM images of the self-healed fabrics: (a) the self-healed boundary of the T-shape structures, (b) the liquified and clamped locations of the fabrics, and (c) the location without UV irradiation and clamped.

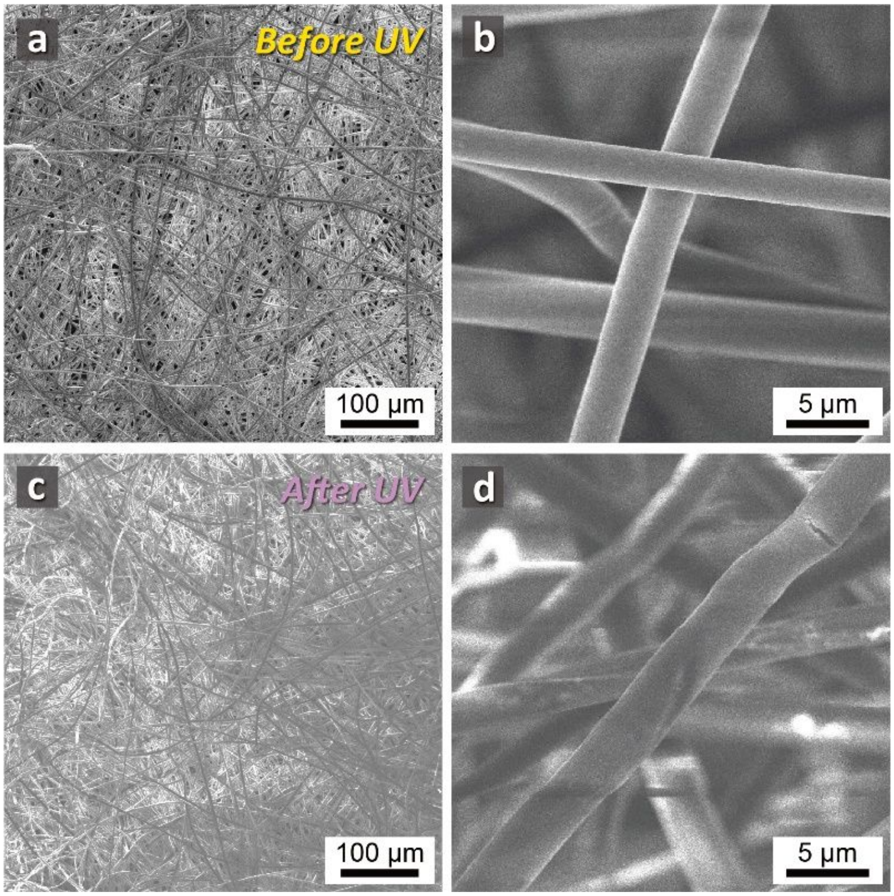

Figure S13. SEM measurements of the PS fibers (a,b) before and (c,d) after exposed to UV light for 24 h.

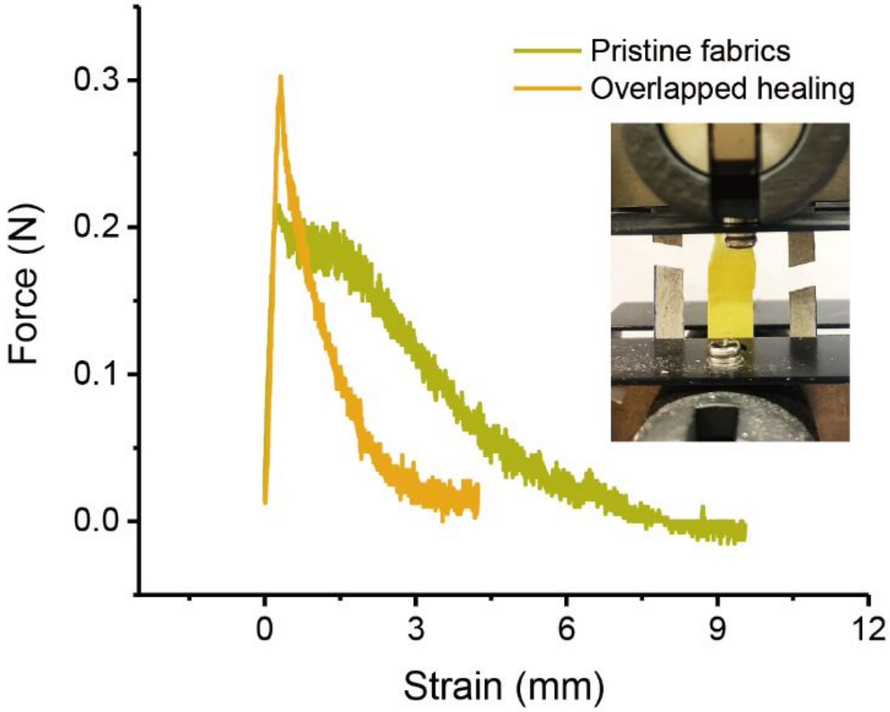

Figure S14. Tensile tests of the pristine and overlapped healing fabrics.
